# Pre-service teachers’ emotional experience: Characteristics, dynamics and sources amid the teaching practicum

**DOI:** 10.3389/fpsyg.2022.968513

**Published:** 2022-09-26

**Authors:** Yilong Ji, Mohamed Oubibi, Siyuan Chen, Yuxin Yin, Yueliang Zhou

**Affiliations:** ^1^College of Teacher Education, College of Education and Human Development, Zhejiang Normal University, Jinhua, China; ^2^Key Laboratory of Intelligent Education Technology and Application of Zhejiang Province, Zhejiang Normal University, Jinhua, China; ^3^Hangzhou Preschool Teacher’s College, Zhejiang Normal University, Jinhua, China

**Keywords:** pre-service teachers', teaching practice, emotional experience, emotional labor, teaching practicum

## Abstract

Recently, teacher emotions have become the focus of research in teacher education. Teacher emotions not only affect teachers themselves but also have an impact on their students. However, pre-service teachers’ emotions have been neglected. This study is based on a qualitative analysis of online emotional diaries related to emotional experience expression by 120 Chinese pre-service teachers before, during, and after teaching practice. The results in this study show three characteristics of pre-service teachers’ emotional experiences: the overall positive emotions are higher than negative emotions; “caring” and “nervous” are the most typical emotions and variability in emotional experience across gender and internship schools. Then, it is surprising that pre-service teachers’ emotional trajectories are complex and dynamic, positive emotions are decreasing, and negative emotions increase as time goes by. Finally, from the perspective of emotional experience sources, organizational factors affect the emotional experience, personal factors, and background factors.

## Introduction

Teaching is an emotional practice (Hargreaves, 2000), and teachers’ emotions are an integral part of their careers, affecting their teaching quality, students’ learning behavior, and academic performance (Sutton and Wheatley, 2003). Although research on teacher education has only focused on rationality and cognition (Day, 2014), more and more scholars have recently begun to consider the field of teacher emotion (Hargreaves, 1998; Zembylas, 2003; Yin, 2016; Chen, 2021). Teacher emotions are currently considered a vital research focus. Firstly, teacher emotions play a crucial role in teachers’ professional beliefs, well-being, and effectiveness (Fried et al., 2015). Secondly, teacher emotions significantly impact learning and teaching (Sutton and Wheatley, 2003; Junjun, 2019). Thirdly, Teacher emotions not only affect teachers themselves but also have an impact on their students (Chen, 2021). And some researchers have begun to pay attention to the emotional problems of pre-service teachers (Uitto et al., 2015; Timoštšuk et al., 2016; Zhu, 2017; Holappa et al., 2021). Pre-service teachers have the dual identities of students and future teachers, and the cultivation of their teaching practice ability has always been an important topic and key link in normal universities (Chen et al., 2011). Pre-service teachers’ emotions are important for teacher burnout, professional identity, teaching effectiveness, and student growth (Bloomfeld, 2010). However, the existing training models for pre-service teachers still focus on applying the knowledge and skills acquired by pre-service teachers in teaching practice. At the same time, ignore the value process of the emotional experience of pre-service teachers (Harris and Sass, 2011). In general, the emotion of pre-service teachers has not received sufficient attention in the field of teacher education research (Bullough and Draper, 2004; Golombek and Doran, 2014). Moreover, there is less research on the emotion of pre-service teachers in the context of Chinese social culture, especially since few studies have explored the emotional experience of pre-service teachers in teaching practice (Liang, 2019; Dong and Chen, 2020).

## Literature review

### Teaching practicum of pre-service teachers

Teaching practicum is a kind of development process to enhance the professional practice ability of pre-service teachers. It concerns teacher educators’ practical application of acquired knowledge, skills and emotions in their teaching practice activities. (Lawson et al., 2015). In many teacher education programs, teaching practicum is a compulsory course to be taken by all the students as it is an important part of a teacher’s professional development (Richards and Crookes, 1988). However, the nature, length and frequency of the practicum vary from one institution to another. Many studies have demonstrated the importance of teaching practicum for developing teacher educators’ teaching skills (Heppner, 1994; Yuan and Lee, 2014). Moreover, teachers’ teaching ability is one of the most important ability factors affecting students’ academic achievement (Nalipay et al., 2019). As the function and status of teaching practicum have gradually come to the fore, it has attracted great attention from the government and scholars.

Although, for a long time, teacher education scholars ignored the value of emotion in teaching practice. Since the late-1990s, More and more scholars are beginning to pay attention to the relationship between teaching practice and emotion (Isenbarger and Zembylas, 2006; Oplatka, 2007; Yin, 2016). Hargreaves (1998, 2000) thinks that teachers’ teaching practice is full of emotion, a very common phenomenon in teaching practice. For example, love, passion, compassion and so on are usually regarded as the most important ethical qualities in the teacher’s professional ethics. Good teaching is often accompanied by interest, pleasure, challenge, creativity, excitement and other emotional factors. Teaching is an emotional work that involves a range of emotions, from joy to anger (Frenzel, 2014). However, normal school students are the object of teaching practice. New teachers get real teaching experience in school through teaching practice. With the deepening of teaching practice, normal students have different emotions in interacting with people or things related to teaching, and emotions are the soul of teaching (Timoštšuk and Ugaste, 2012).

### Characteristics of teachers’ emotional experience

Many studies showed that different types of teachers show different characteristics of emotional experiences. It is usually expressed as positive and negative emotions (Koenen et al., 2019; Taxer et al., 2019). Due to the uncertainty and unpredictability in education and teaching, teachers often experience various emotions, including positive emotions (such as enthusiasm, happiness, and satisfaction) and negative emotions(such as disappointment, anger, and burnout; Nias, 1996; Hargreaves, 1998). Chen (2016) focused on primary school teachers’ emotional experiences and pointed out that happiness, love, sadness, anger, and fear are major dichotomous experiences. In contrast, Xie and Jiang (2021) concentrated on university teachers and revealed that more negative emotions are found, such as anger, enjoyment, anxiety, disappointment, and conflict.

Pre-service teachers play a dual role in teaching practice, both teachers and students. In the process of teaching practice, their emotional experience as novice teacher is often different from that of in-service teachers. Pre-service teachers experience various positive and negative emotions in their interactions with advisors, students, pre-service peers, and other school staff. Timoštšuk and Ugaste’s (2012) research shows that the practice situation or environment will evoke different emotional experiences in pre-service teachers, positive emotions will bring happiness and self-satisfaction, and negative emotions will lead to powerlessness, helplessness, and lack of security. Keller et al. (2014) pointed out that pre-service teachers tend to experience high levels of anxiety, especially when they are unprepared to teach and fail to meet the demands of teaching. Pre-service teachers emotionally experience craving and anxiety at the beginning of a teaching internship, shock and embarrassment after teaching, and guilt and regret at the end of the internship (Zhu, 2017). Most pre-service teachers tend to be full of enthusiasm and self-confidence during their first teaching practice. However, once entering the internship role, one gradually felt great stress and anxiety (Mapfumo et al., 2012) and experienced many negative emotions, such as helplessness, frustration, confusion, embarrassment, and even hostility (Timoštšuk et al., 2016).

### Dynamics in teachers’ emotional experience

Teacher emotion refers to emotion located in a specific environment and evolves; the time factor is also an important content affecting teacher emotion (Zembylas, 2003). Zembylas (2007) believes teachers’ emotions are “a dynamic, constantly fluctuating system of meaningful experiences.” Chen (2017) argues that emotions can be considered dynamic because emotions are fundamentally about sports, and Chinese teachers experience a mix of emotions, first more negative emotions (such as worry and anxiety), then more positive emotions (such as love and happiness). Chen et al. (2020) used a case study method to explore the emotional experiences of two outstanding teachers at different career stages, and the results showed that there were more negative emotions in the early stage, more positive emotions in the middle stage, and higher emotional satisfaction in the later stage. As a representative of outstanding teachers, principals’ research found that principals have more negative emotions than positive ones in their careers and less negative and positive ones in the later stages (Chen and Walker, 2021).

Some studies also show the emotional changes in college students’ practice process. Pre-service teachers emotionally experienced longing and anxiety at the beginning of a teaching internship, shock and embarrassment after teaching, guilt and regret at the end of the internship, and the emotions experienced by pre-service teachers changed over time (Zhu, 2017). Other scholars have studied the impact of emotional trajectories on the formation of pre-service teachers’ professional identity and found that pre-service teachers’ emotional trajectory patterns from the beginning to the end of practice: anticipation and anxiety at the beginning of teaching practice, shock and embarrassment after teaching, and anger during practice and confusion, helplessness and loneliness at the end of the internship, guilt and regret after the teaching practice (Deng et al., 2018). Numerous studies have demonstrated a correlation between changes in emotional experience and the process of professional identification. The identity of novice teachers affects their behaviors and emotions while further influencing the dynamic formation of their professional identity (Thomas and Beauchamp, 2011; Cheng, 2021). Zhu et al. (2020) examined the relationship between perceptions and feelings of professional identity and teaching in student teachers. The results showed that although optimistic initially, anxiety appears as they go through role changes. A better understanding of professional teaching increased with transformational cognitions from different perspectives.

### Sources of teachers’ emotional experience

Emotional experience has a certain orientation and is generated for a certain individual, event, or teaching situation (Gross, 1998). Evidence shows that relations and teaching are major sources of teacher emotional experiences (Hargreaves, 2000; Caires and Almeida, 2005; Timoštšuk and Ugaste, 2012; Wu and Chen, 2018; Yin et al., 2019). Under the context of teacher-student relationships, much of the existing empirical data supports that high student achievement can be a source of positive teaching experience. (Hargreaves, 2000; Beilock et al., 2010; Frenzel, 2014). On the other hand, student misbehavior can be considered one of the key factors in causing negative emotions in teachers, especially anger and anxiety (Hargreaves, 2000; Chang, 2009; Frenzel et al., 2009a,b). Therefore, the change in students always touches the teacher’s heart.

In recent years, more and more researchers have paid attention to teachers’ emotions from the perspective of ecology. Cross and Hong (2012) explained how teachers’ emotions interact with the environment using Bronfenbrenner's (2005) ecological model, which consists of micro-systems, mesosystems, external systems, and macrosystems, exploring how teachers’ internal psychological characteristics interact with the external environment Interact with each other, thereby affecting the production of emotions. Chen (2017) explored the emotional experiences of 53 primary school teachers in the teaching process in Hong Kong and mainland China. These teachers described an equal number of positive and negative emotions. And different types of emotions decrease as the distance from the teachers increases the five nested ecological systems. Teachers experienced fewer emotions within and within the environments further from them. Then, in 2019, Chen used a mixed approach to explore further the emotions of 1,492 primary school teachers in China. Qualitative and quantitative data suggest that high-intensity emotions are present at the micro-system level. Sun and Yang (2021) explored the dynamic emotional development process and characteristics of Chinese high school English teachers in their interaction with the ecosystem and the ecological factors that may affect their emotional development. The results showed that 68 emotions emerged from the two participants: 39 positive emotions and 29 negative emotions. Personal antecedents, contextual antecedents, and teachers’ emotional capacity are the main environmental factors that affect teachers’ emotional development.

The above research focuses on the emotions experienced by teachers when interacting with teaching, students, colleagues, parents, etc. At the same time, few researchers have investigated the emotional experiences of pre-service teachers in teaching practicum. In addition, most of the studies (Chen, 2017; Zhu, 2017; Wu and Chen, 2018) were conducted by qualitative research methods mainly through observation and interviews, which were able to penetrate the actual lives of teachers but had the disadvantages of being less representative and more subjective. Therefore, this study used the content analysis method of the emotional diaries to explore the emotional experiences of pre-service teachers amid the teaching practicum and make recommendations for improving teacher education programs. It is refined into the following research questions:

1. What are the characteristics of pre-service teachers’ emotional experiences during their teaching Practicum?
2. How do pre-service teachers’ emotional experiences change dynamically before, during, and after their teaching Practicum?
3. What are the sources of pre-service teachers’ emotional experiences during their teaching Practicum?

## Materials and methods

### Participant

**Figure 1:**
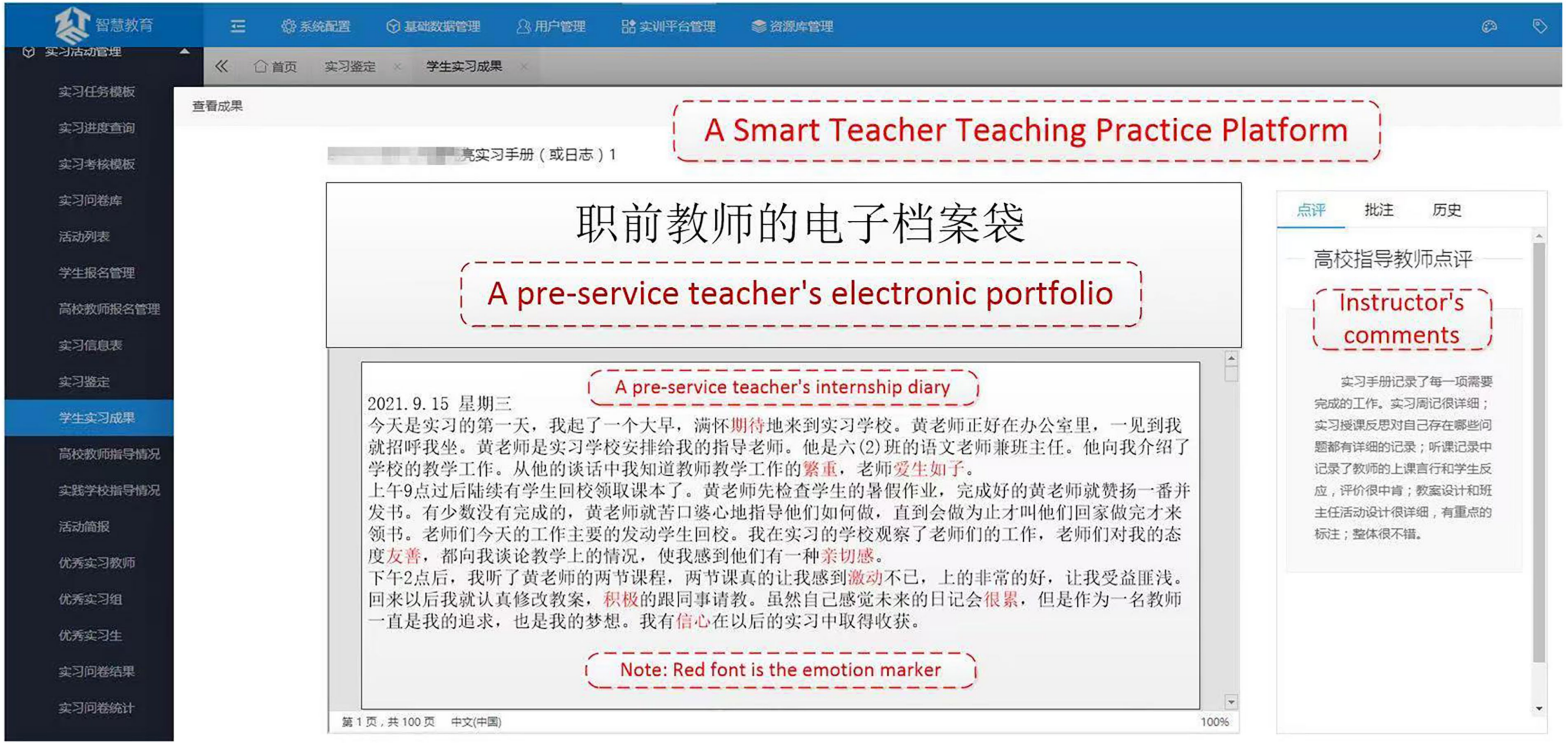
A smart teacher teaching practice platform.

This study was conducted at a Normal University in Zhejiang Province, China, a comprehensive provincial key university with the characteristics of teacher education. The Teaching Practice course is required in the Teacher Education Program at this university. Educational Practice courses are generally offered in the final year of the university. Normal students will take 8–12 weeks to master the basic teaching knowledge and skills. The teaching practice course system’s main contents include classroom teaching, class management, teaching research and home-school cooperation. A university has designed and developed a smart teacher teaching practice platform^1^ that integrates information-based teaching, collaborative management, intelligent evaluation, and data-based research, as shown in Figure 1.

According to the research question, this study adopts the principle of purposeful sampling: selecting informative cases for the study. The nature and substance of these cases will shed light on the research question being investigated (Patton, 2015). A total of 120 normal students were selected to participate in this study. The subjects were informed of the purpose, requirements and precautions of participating in the study to understand their emotional experience in the practice process. The requirement is that each participant upload 3–5 emotional diaries per week, which can be a summary and reflection of the daily classroom teaching. It is important to note that the instructor may give some evaluation and interaction based on the journal entries. Still, these journals are only used for research purposes, do not evaluate the journal’s contents, and will not affect their internship performance. The research objects are 120 pre-service teachers from the internship platform in the three grades of 2017, 2018, and 2019. They are all pre-service teachers who have just completed their teaching practice tasks. Among them, 30% are boys, and 70% are girls. The internship schools are elementary, middle and high schools. The specific distribution of respondents is shown in Table 1.

**Table 1:** Descriptive statistics (*n* = 120).

Gender		Education		Teaching subjects		Practice school		School location	
Male	36 (30%)	Undergraduate	90 (75%)	English	30 (25%)	Primary school	30 (25%)	Rural	22 (18%)
Female	84 (70%)	Master	30 (25%)	Chinese	60 (50%)	Middle school	60 (50%)	County	38 (32%)
				Math	30 (25%)	High school	30 (25%)	City	60 (50%)

### Data collection and analysis

This study conducts a qualitative study on the electronic emotional diaries of 120 pre-service teachers. The emotional diary is not only a cognitive behavior of teachers but also accompanied by teachers’ emotional experience in the process, which is a process in which reason and emotion are intertwined. It is regarded as a special form of the questionnaire. The subjects in the diary can regularly record emotional events in different time stages in detail, which can be used as the original material for emotional analysis. Sometimes diaries are more suitable for dealing with teachers’ emotional sensitivity than interviews. Compared with the interview, it avoids subjectivity. It has more details, especially the detailed response to emotional events, which can make the research focus more on the change process of emotion (Gronn and Lacey, 2004; Yin, 2016). Additionally, emotional diaries give participants enough time to reflect on their major emotional experiences during their teaching practice (Deng et al., 2018).

**Figure 2:**
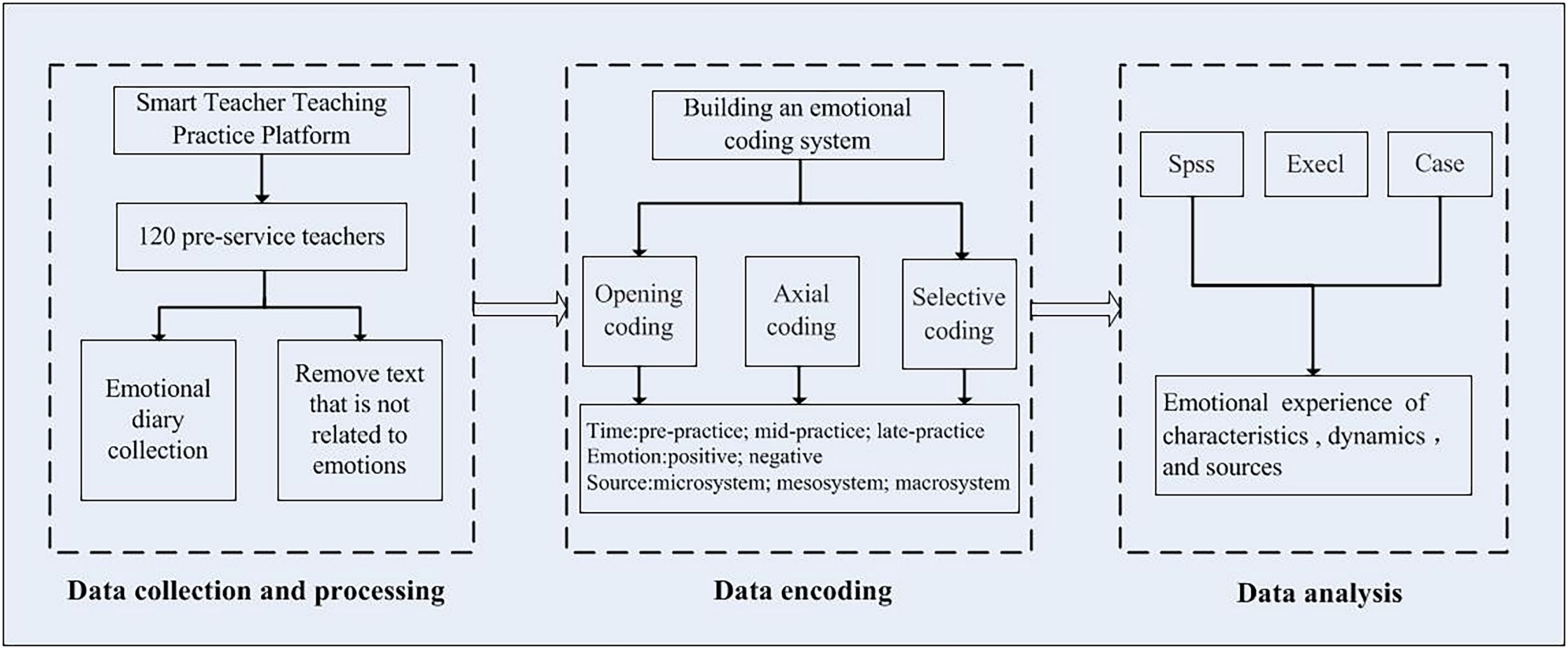
A diagram of the research process.

The research process of this study is shown in Figure 2, which is divided into three steps: data collection and processing, data coding, and data analysis.

The participants were asked to upload three to five journal entries a week as part of an electronic portfolio of internships. The researchers spent 1–4 weeks (pre-practice) and 5–8 weeks (mid-practice). In three different stages of 9–12 weeks (Late-practice), each stage randomly selected two emotional diaries to represent their emotional status at this stage. Therefore, each normal school student collected a total of six emotional diaries. After removing irrelevant records and textual content that only involves emotional experience, and filtering expressions related to emotional experience, a total of 638 diaries were involved in coding, with a total of 286,152 Chinese characters.

Then, two graduate students used the qualitative analysis software Nvivo12 to process and encode the 638-text data collected. They were trained in three steps of coding (Gioia et al., 2013; Aslam et al., 2021). (The coding process is shown in Table 2). The coding scheme is based on the Positive and Negative Affect Scale PANAS (Watson et al., 1988). It is a globally popular and well-validated scale for measuring emotion which includes 10 positive emotions (Interested, Excited, Strong, Enthusiastic, Proud, Alert, Inspired, Determined, Attentive, Active) and 10 negative emotions (Irritable, Distressed, Upset, Guilty, Scared, Hostile, Ashamed, Nervous, Jittery, Afraid).

**Table 2:** A sample of data coding steps.

**Research Subjects**	**Opening coding**	**Axial coding**	**Selective coding**
Zhang Hong	2021.9.16: The expectant eyes of my students and their liking for me made me experience the joy of being a teacher and made me very determined to choose teaching as a career.	Week1 Determined Teacher Belief	Pre-practice Negative emotions Microsystem
Li Ming	2021.10.8: I am very happy that the discipline of this class is very good, and the students are very attentive to the lesson, and the students’ correct rate of the exercise homework after the lesson is relatively high.	Week5 Happy Students’ good performance	Mid-practice Positive emotions Mesosystem
Sun Lin	2021.11.2: Nowadays, if teachers criticize students a little harshly, parents, media, society and experts may criticize teachers, and this social pressure makes me very anxious.	Week7 Anxious High expectations of society	Late-practice Negative emotions Macrosystem

The underlined parts refer to teachers’ emotions. The highlighted parts refer to the source of the emotional experience.

Further, researchers combine the specific content of the text and try to use the constructed emotional dictionary for coding. If no sentiment word in the text content matches the code, two experts are consulted to determine a new emotional label. A total of 12 new emotional labels were found in this study, which were caring, happy, satisfied, grateful, expectant, calm, tired, sad, helpless, stressful, frustrated, and worried. This may result from the emotional characteristics of pre-service teachers and their Chinese background. After the transcription is completed, two experts verify the code’s validity.

Finally, researchers used various software such as SPSS 24.0 and excel 2022 to analyze the data and interpret the cases in the emotion diary to understand the characteristics of preservice teachers’ emotional experience, visualize the trajectory map of preservice teachers’ emotional experience from the beginning to the end of practice, and establish a model of the influencing factors of preservice teachers’ emotional experience.

## Findings

### Characteristics of the emotional experience of pre-service teachers

Through qualitative statistics and analysis of 638 online emotional diaries submitted by pre-service teachers in teaching practice, the qualitative data found that in the process of teaching practice, pre-service teachers’ emotional experiences are characterized by the following:

First, the overall positive emotional experience is higher than the negative emotional experience. Since each text material may have multiple emotional experiences, a total of 4,615 emotional tags were obtained, each emotional diary had an average of 7 emotional tags, and each pre-service teacher had an average of 38 emotional tags. There are as many as 30 types of emotional experience; positive and negative emotions are almost equal, 15 are positive, and 15 are negative. An overall number of most emotional experiences are positive emotions, with 2,803 positive emotions accounting for 61% and 1,812 negative emotions accounting for 39%.

Second, “caring” and “nervous” are the most typical emotions in pre-service teachers’ teaching practices. The emotional experience label of each teacher would appear multiple times. The data is screened for a second time to remove the repeated records of a certain emotional experience of each pre-service teacher to ensure that a certain emotional word appears only once in a sample of pre-service teachers. After the secondary screening of qualitative data, a summary table of the frequency and proportion of the overall emotional experience of Chinese pre-service teachers is obtained, as shown in Table 3.

**Table 3:** Percentage of pre-service teachers who reported each emotional experience.

CPST-PANAS(*N* = 120)					
PA	Frequency	Proportion	NA	Frequency	Proportion
Caring	95	79%	Nervous	82	68%
Happy	78	65%	Tired	61	51%
Excited	70	58%	Distressed	56	47%
Satisfied	64	53%	Sad	52	43%
Enthusiastic	58	48%	Irritable	45	38%
Proud	55	46%	Helpless	42	35%
Grateful	49	41%	Afraid	38	32%
Active	37	31%	Stressful	29	24%
Interested	33	28%	Upset	18	15%
Determined	31	26%	Jittery	15	13%
Expectant	28	23%	Ashamed	10	8%
Inspired	21	18%	Frustrated	7	6%
Strong	13	11%	Worried	5	4%
Alert	8	7%	Hostile	4	3%
Calm	5	4%	Guilty	3	3%

Visualize from the table that 79% of the pre-service teachers felt the emotion of “caring.” The main principle of education is love. Love is one of the main positive emotions experienced by humans (Oehman, 2008). Pre-service teachers must maintain love in student growth and express love in getting along with colleagues. Caring is pre-service teachers’ primary positive emotional experience, the core of teachers’ professional emotions, and an eternal education topic. However, 68% of pre-service teachers feel “nervous” during their teaching practice. Nervous has been the most widely studied effective problem in human emotion since the 1990s (Horwitz, 1996). For pre-service teachers, students’ expectations, lack of subject knowledge and teaching experience are all causes of tension when they step on the podium for the first time. Facing the conflict between reality and ideals, pre-service teachers struggle with the relationships between teaching, students and colleagues. Nervous is the primary negative emotional experience of pre-service teachers, and nervousness significantly affects teachers’ self-efficacy, job burnout and occupational well-being (Han et al., 2020).

There is some variability in the emotional experiences of pre-service teachers. In this study, different types of pre-service teachers’ emotional experience net effect were tested, the results are shown in Table 4. The net effect of affective experience is the average of the difference between the number of positive and negative emotions reported by each teacher. If the average of this difference is positive, the teacher reports more positive emotions than negative ones. If it is negative, the instructor reports more negative emotions than positive ones. The results show that, there are significant differences in gender and practice school. The difference is that the net emotional experience of female pre-service teachers is −0.623, while that of male pre-service teacher is 1.152 (*t* = 6.152, *p* = 0.000 < 0.001), indicating that female pre-service teachers reported significantly higher amounts of negative emotions than male preservice teachers. In addition, the net emotional experience of pre-service teachers who interned in elementary school compared to middle and high school was 1.513 (*t* = 3.264, *p* = 0.025 < 0.05), indicating that the number of positive emotions reported by pre-service teachers in primary schools was significantly higher than that reported by negative emotions. However, the emotional experience did not differ significantly in educational background, subjects taught and school location.

**Table 4:** Results of the test of variability of the positive means.

**Type**	**Specific division**	**M (PA-NA)**	**SD**	**F/t**	**P**
Gender	Male	1.152	0.693	6.152	0.000^***^
	Female	−0.623	0.552		
Education	Undergraduate	1.045	0.724	1.866	0.647
	Master	0.574	0.573		
Teaching Subjects	English	−0.283	0.215	1.375	0.108
	Chinese	0.361	0.472		
	Math	0.827	0.851		
Practice School	Primary school	1.513	0.904	3.264	0.025^*^
	Middle school	0.356	0.672		
	High school	−0.138	0.138		
School Location	Rural	0.674	1.206	1.548	0.271
	County	0.245	0.734		
	City	0.129	1.032		

M is the mean, SD is the standard deviation, T is the value of the t-test statistic, and P refers to the probability of that outcome or more extreme cases when the original hypothesis is true.

* *p < 0.05;*

*** *p < 0.001.*

### Dynamics in the emotional experience of pre-service teachers

**Figure 3:**
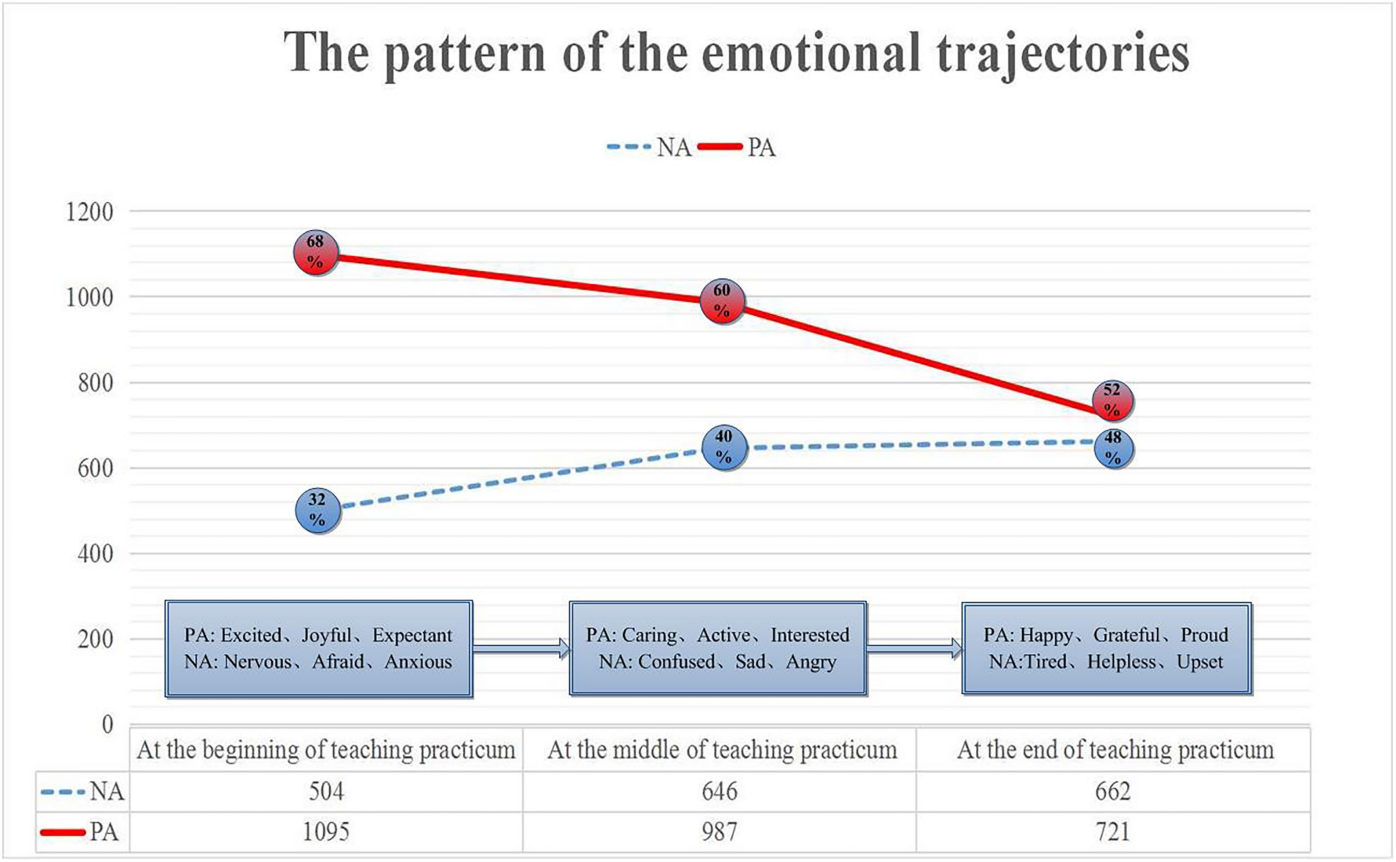
Emotional trajectory pattern of pre-service teachers in the process of teaching practice.

The teaching practice of Chinese pre-service teachers generally takes about 12 weeks of intensive practice. The researchers divided the internship stage into the pre-internship period (the first 4 weeks), the middle period of the internship (the middle 4 weeks), and the late period of the internship (the last 4 weeks) according to the time sequence of the internship—emotional labels for stages. Through statistical analysis of quantitative data, it is found that pre-service teachers have complex, diverse and changing emotional experiences in different stages of teaching practice. To further visualize the changes in the emotional characteristics of the pre-service teachers, the emotional labels in the Nvivo12 matrix were imported into excel, and the pre-service teachers’ emotional experience trajectory pattern in the teaching practice process was obtained, as shown in Figure 3.

Figure 3 shows intuitively that from the overall tendency, pre-service teachers show changes in different emotional experiences at different stages of teaching practice. Positive emotional characteristics are more than negative emotional characteristics in different stages, indicating the overall emotional experience of pre-service teachers active and focused. From the perspective of emotional changes, the number of positive emotional features gradually decreased with time, and the number of negative emotional features increased steadily. In the early stage, the expressions of positive emotional experience were the most abundant and varied, accounting for 68%, such as excited, joyful, and expectant, with fewer negative emotional experiences, such as nervousness, afraid, anxiety, etc. In the mid-term, the positive emotional experience slightly decreased but still accounts for 60%, such as caring, active, interested, and inspired. The negative emotional experience increased significantly, accounting for 40%, such as confused, sad, and angry. Later, the positive emotional experience further declined, and the proportion dropped to 52%. It mainly focuses on emotional experiences such as happiness, gratitude, pride, and persistence. The number of negative emotional experiences decreased slightly and stabilized, but the proportion increased significantly, reaching 48%. Mainly focus on negative emotions such as tiredness, helplessness, disappointment, and frustration.

### The source of the emotional experience of pre-service teachers

This study refers to the teacher emotional ecosystem model Cross and Hong (2012) proposed. However, due to the particularity of pre-service teachers and the background of Chinese culture, we have adjusted the system and content of the division. Because the emotional experience of pre-service teachers is highly contextual, when dividing different systems, the work field of pre-service teachers should be fully considered. The elements in the work field (such as students, peers, instructors, and leaders) should be compared with work Elements outside the field (e.g., family, friends) should be closer to the inner circle. Therefore, this study regards the individual factors that affect the emotional experience of pre-service teachers as a micro-system, incorporates the elements of pre-service teachers’ interaction in the workplace into the mesosystem, and finally classifies social culture and educational reform as a macro-system.

This research uses the emotional dictionary to carry out a secondary coding analysis of all the text materials with the emotional experience of the pre-service teachers according to the emotional orientation factors. It explores the specific orientation of the pre-service teachers in terms of emotional experience. Combining the existing literature and text analysis, the researchers summarized three first-level dimensions, including Micro-system (personal factor), Mesosystem (organizational factor), and Macrosystem (contextual factor). There are 9 secondary dimensions: emotional intelligence, teacher professional identity, teacher belief, student, instructor, internship peer, school leader, social culture, and educational reform (see Table 5).

**Table 5:** Sources of the emotional experience of pre-service teachers.

First dimension	Second dimension	Frequency of Occurrence (*N* = 4,615)	Total Proportion (%)	Positive affect frequency (*N* = 2,803)	Proportion of positive emotions (%)	Frequency of Negative Emotions (*N* = 1,812)	Percentage of negative emotions (%)
Microsystem (Personal reason)	Emotional intelligence	320	6.9%	185	56.9%	135	43.1%
	Teacher professional identify	281	6.1%	154	54.8%	127	45.2%
	Teacher belief	402	8.7%	264	65.7%	138	34.3%
Mesosystem (Organizational factor)	Student	1,610	34.9%	990	61.5%	620	38.5%
	Instructor	821	17.8%	654	79.7%	167	20.5%
	Internship peer	566	12.3%	316	55.8%	250	44.2%
	School leader	195	4.2%	75	38.5%	120	61.5%
Macrosystem (Backgroun factor)	Social culture	258	5.6%	114	44.1%	144	55.9%
	Education reform	162	3.5%	51	31.5%	111	68.5%

#### Micro-system (personal factors)

From the perspective of personal factors, the frequency of emotional experience points to personal factors as high as 1,003, accounting for 21.7% of all emotional experiences. It is a secondary factor affecting emotional experience. Qualitative data shows that the positive emotions reported by pre-service teachers at this level are higher than those of negative emotions, and the proportion of positive emotions reaches 60.1%. Specific to the second dimension, the frequency of emotional experience points to teachers’ beliefs reached 402, accounting for 8.7%, of which the proportion of positive emotions was significantly higher than that of negative emotions, reaching 65.7 and 34.3%, respectively. The frequency of emotional experience points to emotional intelligence reached 320, accounting for 6.9%, of which the proportion of positive emotions was higher than that of negative emotions, reaching 56.9 and 43.1%, respectively. The frequency of emotional experience points to teacher professional identifies reached 281, accounting for 6.1%. The proportion of positive emotions was slightly higher than that of negative emotions, reaching 54.8 and 45.2%, respectively. Positive affective experiences involving self-confidence, pride, persistence, and serenity expressed positive feelings of pre-service teachers about teacher belief, emotional intelligence, and professional identity. At the same time, it also includes negative emotional experiences such as frustration, fear, and depression, expressing negative feelings about issues such as teacher role, self-efficacy, and professional identity. For example, a pre-service teacher wrote in his emotional diary:

"Today will be the first time I stand on the stage as a teacher. Although the content of the classroom teaching is only to explain the test paper I practiced last week, and the internship instructor Zhang also encouraged me, I still feel very nervous. After installing the microphone of the little bee amplifier, I hurriedly put it on my shoulders, holding the textbook in one hand and the paper in the other. Although I don't know if the students understood what I said, I saw their serious expressions and answers of the students. This positivity gives me full confidence in my future teacher life.” (T1-C3-E16-M1)

The above diary describes the pre-service teacher’s influence on the production of the teacher’s emotional experience due to the difference in emotional ability. Studies have shown that a teacher with poor emotion regulation skills often produces negative emotions such as tension, fear, and sadness during the teaching process and may have a stronger response to negative emotional events (Grandey, 2000).

#### Mesosystem (organizational factors)

From the perspective of organizational factors, the frequency of emotional experience-oriented organizational factors is as high as 3,192, accounting for 69.2% of all emotional experiences. It is the primary factor affecting emotional experience. Qualitative data showed that the positive emotions reported by pre-service teachers at this level were significantly higher than the number of negative emotions, with the proportion of positive emotions reaching 63.8%. Specific to the second dimension, the frequency of emotional experience points to students reached 1,610, accounting for 34.9%, and the proportion of positive emotions reached 61.5%. Emotional experience points to instructors appearing 821, accounting for 17.8%, and positive emotion accounting for 79.7%, the first factor affecting positive emotional experience. Emotional experience points to the frequency of internship peers appearing 566, accounting for 12.3%, and the gap between positive emotions and negative emotions is not large. Emotional experience points to the frequency of school leaders appearing 195, accounting for 4.2%, and the proportion of negative emotions is significantly higher than that of positive emotions. Positive emotional experience mainly comes from the care of “students,” the gratitude of “instructors,” and the pleasure of “internship companions.” For example, a pre-service teacher wrote in his emotional diary:

"Internship is over. The night before I left, the students gave me a huge surprise. They sang for me together, and I burst into tears. They also made me a greeting card that the whole class signed. This night was a totally moving night." (T3-C101-E7-M4)

The above diary describes the most caring, satisfying and enthusiastic teacher-student relationship between the pre-service teacher and the student. The teacher-student relationship is a kind of interpersonal relationship that reflects the psychology and behavior of the pre-service teachers and students in the school interacting with each other, interacting with each other, and influencing each other. The reason is that as new teachers, pre-service teachers often forget their teacher status and conduct equal dialogues and exchanges with students based on equal interpersonal communication so that most positive emotional experiences can be obtained during the short internship period.

However, the negative emotional experience mainly comes from anger at “students’ bad behavior,” the hostility of “internship peers,” and disappointment with “school leaders.” For example, a pre-service teacher wrote in his emotional diary:

"Some students will engage in small movements during class, drink drinks during English dictation, and keep touching their hair and jewelry when you are not staring at them. But to maintain the efficiency of the whole class, they also with quiet discipline, I can only deliberately raise my voice to remind them to keep quiet. If these things don't get better, it makes me feel angry to see them not fully engaged in learning." (T2-C95-E20-M4)

The above diary describes that the pre-service teachers have many negative emotions in the face of students’ bad behavior, such as: expressing anger, confusion, frustration and other negative emotional experiences in situations such as controlling classroom discipline, non-teaching work and student management. Surprisingly, many pre-service teachers described the negative emotions of competition with their peers. Some pre-service teachers felt helpless when the pre-service school leaders assigned them heavy non-teaching tasks.

#### Macrosystem (contextual factors)

From the perspective of background factors, the frequency of emotional experience points to background factors is only 420, accounting for only 9.1% of all emotional experiences. Qualitative data show that the negative emotions reported by pre-service teachers at this level are significantly higher than the number of positive emotions, with negative emotions accounting for 60.7%. Specific to the second dimension, the frequency of emotional experience points to social culture reached 258, and the proportion of negative emotions reached 55.9%. The frequency of emotional experience points to education reform reached 111, and negative emotions accounted for 68.5%, the first factor affecting negative emotional experience. Positive emotions are associated with a social climate of respect for teachers, including happiness, caring, satisfaction, and excitement. Negative emotions are related to unrealistic educational reforms and social prejudice against teachers, often manifested as confusion, helplessness and anxiety. For example, a pre-service teacher wrote in his emotional diary:

"The school where I practiced is implementing the "double deduction" policy, which requires the school to break the concept of only recognizing marks, strictly prohibit ranking and grading students, and prohibiting test questions that exceed the standard. Special emphasis is also placed on improving the quality of school assignments, requiring teachers to design hierarchical, flexible, and personalized assignments. But I found that there are still many problems in the specific implementation process. For example, the phenomenon of ranking is still very common in our school. Although we do not publish rankings to students, parents still ask about their children's specific rankings. There are 40 students in the class, and if I have 40 personalized assignments to do, I don't have enough energy.” (T2-C26-E21-M9)

The above diary describes the negative emotions of pre-service teachers when facing the implementation of education reform. The “double reduction” policy is that on July 24, 2021, the General Office of the Central Committee of the Communist Party of China and the General Office of the State Council issued the “On Further Reduction in Students’ Homework“Compulsory Education.” The Opinions on the Burden of Homework and the Burden of Off-campus Training” calls for reducing the burden of homework and off-campus training for students. Many pre-service teachers express enthusiasm and positive emotions of approval for the intensive and unrealistic educational reform measures but often feel helpless and ambivalent under pressure, mainly because the education reform did not consider the actual situation. Pre-service teachers only know what educational reform is, not why and how.

#### Model of influencing factors of pre-service teachers’ emotional experience

**Figure 4:**
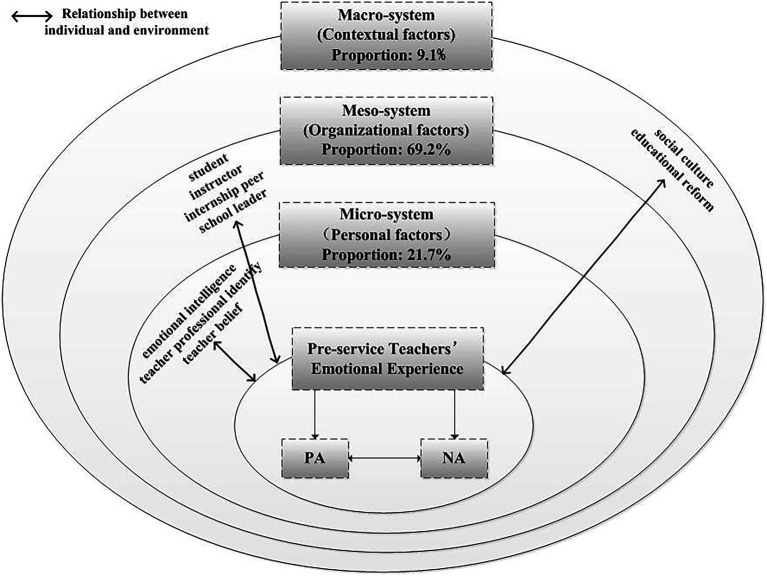
Revision map of influencing factors of pre-service teachers’ emotional experience.

According to the above analysis, it is concluded that the influencing factors of the emotional experience of pre-service teachers mainly include three levels: micro-system, mesosystem and macro-system. First, the micro-system (personal factors) is mainly reflected in three aspects: emotional intelligence, identity, and teacher beliefs. Emotional intelligence refers to the ability to recognize and use emotional information in social interactions (Grandey, 2000), and effective emotional regulation affects the specific performance of teachers’ emotions. Pre-service teachers have a special identity in teaching practice schools. They have to play the role of both teachers and students. Their different sense of identity as teachers affects the different emotional experiences of teachers. Teacher belief is an inner concept and implicit assumption teachers hold about teacher role, teacher-student relationship, teaching and learning (Kagan, 1992). It is generally believed that teachers with strong educational beliefs express more positive emotions. Secondly, the mesosystem (Organizational factors) is mainly reflected in four aspects: students, internship peers, instructors and school leaders. Students are the most closely interacting objects of pre-service teachers in the process of teaching practice. Students’ progress can be the main source of teachers’ positive experiences, and students’ bad behavior can be the main source of teachers’ negative emotions. (Hargreaves, 2000; Beilock et al., 2010; Frenzel, 2014). In collective teaching practice, pre-service teachers often share their true feelings. When they feel confused or helpless, they will help each other and seek common understanding and comfort, but they are also surprised to find that they are hostile due to competition of negative emotions. Mentors play a vital role in the professional growth of interns, helping them acquire knowledge and skills that cannot be learned on their own (Hawkey, 1998; Bullough and Draper, 2004; Hobson et al., 2009). The interaction between instructors and pre-service teachers directly affects the quality of the emotional experience. Thirdly, the macro-system (Contextual factors) is mainly reflected in two aspects: social culture and educational reform. The traditional Chinese teacher culture pays attention to the social nature of teachers and endows teachers with more social responsibility and social image. The social atmosphere of respecting teachers and respecting morality can promote the positive emotional experience of pre-service teachers. As educational reform brings more responsibilities and tasks, many teachers show negative emotions of dissatisfaction, helplessness, stress and anxiety (Lee et al., 2013). According to the teacher’s emotional ecosystem model proposed by Cross and Hong (2012) and the coding and analysis of the above qualitative data, the model of influencing factors of pre-service teachers’ emotional experience was revised, consisting of a micro-system, mesosystem, and macro-system specifically as shown in Figure 4.

## Discussion

### Characteristics of the emotional experience of pre-service teachers

This study identified three characteristics of preservice teachers’ emotional experiences in their teaching practicums. First, it is reassuring that the overall positive emotional experience of the pre-service teacher is higher than the negative emotional experience. This finding is different from the previous emotional experience of in-service teachers. Such as Chen’s (2017) qualitative research found that 53 teachers described 68 kinds of emotions. These included 34 positive emotions (e.g., happiness, caring, pride) and 34 negative emotions (e.g., pressure, sadness, worry). Teachers in Hong Kong and the Chinese mainland describe a high proportion of negative emotions (50%). Wu and Chen (2018) interviewed 28 primary school teachers in Hong Kong and found that these teachers described 78 emotions, including 40 positive emotions (51.3%) and 38 negative emotions (48.7%). This result may be that in the process of teaching practice, pre-service teachers stand on the podium for the first time, lack experience and teaching in emotional education, and due to the short duration of the internship, they do not have the same experience and rich emotional experience as experienced teachers. These reasons may have caused pre-service teachers to have more positive emotional experiences than in-service teachers (Yang, 2019).

Second, Caring is the primary positive emotion in the teaching practice process of pre-service teachers. This is the same as Junjun (2019) found that primary school teachers’ most common positive effect is Caring. Chinese teachers’ professional standards require teachers to have love, and love is the core of teachers’ professional emotions. Traditional Chinese culture emphasizes that teachers should care about students and maintain a good teacher-student relationship with students (Yin, 2016). The primary negative emotion of pre-service teachers is Nervous, which is different from the primary negative emotion of mature teachers. Still, it is verified that Nervous is a relatively special and complex psychological state caused by the body and spirit when faced with uncertain factors. Pre-service teachers and young teachers are more likely to be victims of Nervous (Sutton and Wheatley, 2003; Chang, 2009). This phenomenon may be that most pre-service teachers set foot on the podium for the first time. Due to their lack of teaching experience, they have high expectations for themselves and often experience tense emotional experiences in the face of real teaching situations.

Finally, there is some variability in the emotional experiences of pre-service teachers. Female pre-service teachers showed more emotional experience, and most of them were negative emotions. The pre-service teachers in primary school showed more emotional experience, and most were positive emotions. Many studies have shown that individual differences lead to differences in emotional experiences (Zhao and Zhang, 2017; Junjun, 2019). Female teachers are generally considered more sensitive, have more volatile mood swings, and are likelier to feel subtle negative emotions (Yang, 2019). In addition, compared with junior and senior high school teachers, primary school teachers are often classified as “high emotional labor” professionals by scholars (Kang, 2022).

On the one hand, primary school is a critical period of children’s emotional development. However, the pupils’ emotions are unstable, rich and personalized, which leads to more diverse emotional experiences. On the other hand, elementary school teachers need to interact with students and parents, colleagues and administrative leaders. This kind of multiple, complex and frequent interpersonal interaction is more than other advanced teachers.

### Dynamics in the emotional experience of pre-service teachers

In addition, what is surprising is that the pre-service teachers found that the emotional experience of the pre-service changed dynamically, the positive emotional characteristics gradually decreased, and the proportion decreased month-on-month. In contrast, the negative emotional characteristics increased steadily, and the proportion increased month-on-month. The teaching practice did not increase the positive emotions of pre-service teachers but brought more negative emotions. These conclusions further validate some studies that teachers’ emotions are complex and dynamic (Harper, 2012; Anttila et al., 2016). This further supports the findings of Bloomfeld (2010) that the process of learning and teaching for pre-service teachers is changing, full of excitement, joy, and even confusion, and it is the process of teacher professional development. Sun and Yang (2021) revealed that the emotions of Chinese English teachers changed significantly over time. They evolved from positive to negative, and finally, mainly positive. Zhu's (2017) study of seven pre-service teachers showed that pre-service teachers experience a range of emotions ranging from excitement to disappointment. At the beginning of the internship, all seven participants were expected and excited about their internship teaching, and after the internship teaching, they were surprised and confused. With the deepening of teaching practice, the positive emotions of pre-service teachers gradually decreased, and the negative emotions increased significantly. The changes in emotional experience also verified that teaching practice is a process of changing from new to in-service teachers. It also verifies the previous research conclusion that in-service teachers have more negative emotions than new teachers (pre-service teachers), and there is a correlation between emotional trajectories and professional identity processes (Zembylas, 2005; Yuan and Lee, 2016; Deng et al., 2018). Teaching practice can enhance pre-service teachers’ professional identity and self-efficacy (Timoštšuk and Ugaste, 2012; Zhang et al., 2016; Zhao and Zhang, 2017).

### The source of the emotional experience of pre-service teachers

In summarizing these findings on sources of pre-service teachers’ emotional experience, it is concluded that the influencing factors of the emotional experience of pre-service teachers mainly include three levels. The first is the organizational factor, followed by the personal and contextual factors. It is particularly important to note that school leadership, social culture and educational reform are the main factors influencing the generation of negative feelings. This finding is consistent with Yin and Li's (2011) findings that teachers are experiencing high stress and anxiety during educational reforms. Chen (2016, 2017; Junjun, 2019) also concluded that teachers’ negative emotions are related to the frequency of educational reforms and teachers’ social expectations. Surprisingly, pre-service teachers can conflict with the authority of school leaders, which can lead to negative emotions. Many pre-service teacher’s express dissatisfaction with the non-teaching tasks assigned by school leaders. They spend a lot of time in a large number of offices. Work, believing that these jobs have nothing to do with teaching jobs, often makes them angry and confused (Deng et al., 2018). Likewise, early-career teachers are distressed by the authority of school leaders because they think they spend a lot of time in non-teaching jobs (Tsang and Kwong, 2016).

## Conclusion and implications

The teaching practice of pre-service teachers is emotional labor, which involves a series of emotional experiences (Hargreaves, 1998). Previous research has focused more on in-service teachers’ emotions. This study is based on a qualitative analysis of online emotional diaries related to emotional experience expression by 120 Chinese pre-service teachers at different stages of teaching practice. The result suggests optimizing pre-service teachers’ emotional experiences, not only for teachers themselves but also for future teacher education programs.

First, in contrast to in-service teachers, preservice teachers’ emotions have their characteristics in teaching practice. As new teachers, pre-service teachers have more positive emotions and are full of yearning and expectations for their future teaching careers. Teaching practice is the initial stage of a teacher’s career. The emotional experience in this stage plays a key role in the teacher’s role identification and the willingness to continue to be a teacher. Therefore, we should not ignore the emotional experience of pre-service teachers but should actively protect their positive and valuable emotional state while carrying on the educational pursuit of student development. Special attention must be paid to female and primary school teachers, who are considered to have experienced more emotional experiences and need more emotional support.

Second, with the deepening of teaching practice, positive emotions are decreasing, and negative emotions are increasing. This is because current teacher training institutions do not offer special courses in emotional education and neglect instruction in whole-process teaching practice. Therefore, teacher training institutions need to reconstruct the curriculum system of emotion education, add courses on emotion education and negative emotion regulation, and build a platform for whole-process teaching practice. Using these platforms to incorporate emotional education into the whole teacher education process will help pre-service teachers obtain emotional education support at different needs, stages, and locations.

Third, since most emotions occur in the mesosystem, organizational factors (students, internship peers, instructors and school leaders) influence the emotional experiences of pre-service teachers. Therefore, multiple forces need to be connected and integrated to strengthen the emotional support of the organization. Instructors should not only pay attention to the emotional confusion of pre-service teachers in daily practice but also formulate an emotional handbook for educational practice to provide more emotional guidance for the professional development of pre-service teachers as “educational status.” Internship peers should build an internship community and establish a sustainable, trusting, mutually beneficial interpersonal relationship and organizational atmosphere. Establishing a two-way mutual emotional communication mechanism between teachers and students is necessary. Furthermore, it is particularly emphasized that school leaders should actively create a warm and caring school atmosphere, reduce the non-teaching burden of pre-service teachers, and regulate the emotional expression of pre-service teachers so that emotional expression can be followed by rules and the occurrence of negative emotions can be reduced.

Although some seminal findings were obtained in this study, there are some limitations to this study, as the small sample of teachers and the exploratory qualitative analysis precluded generalization beyond the scope of this study. Although the most widely used method in studying teachers’ emotions is to obtain information directly from the teachers themselves, this is usually done through a one-time retrospective self-report, either qualitative (interviews) or quantitative (questionnaires) (Frenzel, 2014). Unfortunately, recall-based emotion ratings do not represent emotions’ true frequency and intensity in real life. Therefore, subsequent researchers could use new methods to explore teachers’ emotions, such as using video analysis to observe teachers’ emotional performance in the classroom or capturing teachers’ physiological signals during teaching, which would provide a more direct and objective measure of teachers’ emotions.

## Data availability statement

The original contributions presented in the study are included in the article/supplementary material, further inquiries can be directed to the corresponding author.

## Ethics statement

Ethical review and approval was not required for the study on human participants in accordance with the local legislation and institutional requirements. The patients/participants provided their written informed consent to participate in this study.

## Author contributions

YJ: writing original draft, conceptualization, data curation, and software. MO: writing original draft, conceptualization, methodology, and software. SC and YY: review and editing. YZ: project administration, visualization, and resources. All authors contributed to the article and approved the submitted version.

## Funding

This work was supported by Key Research and Development Program of Zhejiang Province, Grant/Award Number: 2021C03141 and 2022C03106. Project supported by Open Research Fund of College of Teacher Education, Zhejiang Normal University (No. jykf22026).

## Conflict of interest

The authors declare that the research was conducted in the absence of any commercial or financial relationships that could be construed as a potential conflict of interest.

## Publisher’s note

All claims expressed in this article are solely those of the authors and do not necessarily represent those of their affiliated organizations, or those of the publisher, the editors and the reviewers. Any product that may be evaluated in this article, or claim that may be made by its manufacturer, is not guaranteed or endorsed by the publisher.

## Footnotes

^1^ http://www.uteacher.com/
